# Mercaptoalbumin Is Associated with Graft Patency in Patients Undergoing Coronary Artery Bypass Grafting

**DOI:** 10.3390/antiox11040702

**Published:** 2022-04-02

**Authors:** Maura Brioschi, Erica Gianazza, Daniele Andreini, Saima Mushtaq, Laura Cavallotti, Fabrizio Veglia, Calogero C. Tedesco, Gualtiero I. Colombo, Mauro Pepi, Gianluca Polvani, Elena Tremoli, Alessandro Parolari, Cristina Banfi

**Affiliations:** 1 Centro Cardiologico Monzino IRCCS, 20138 Milan, Italy; maura.brioschi@ccfm.it (M.B.); erica.gianazza@ccfm.it (E.G.); daniele.andreini@ccfm.it (D.A.); saima.mushtaq@ccfm.it (S.M.); laura.cavallotti@ccfm.it (L.C.); fabrizio.veglia@gmail.com (F.V.); calogero.tedesco@ccfm.it (C.C.T.); colombo.gualtiero@ccfm.it (G.I.C.); mauro.pepi@ccfm.it (M.P.); etremoli@gvmnet.it (E.T.); 2 Department of Biomedical and Clinical Sciences “Luigi Sacco”, University of Milan, 20157 Milan, Italy; 3 Cardiovascular Tissue Bank of Milan, Centro Cardiologico Monzino IRCCS, 20138 Milan, Italy; gianluca.polvani@ccfm.it; 4 Department of Clinical Sciences and Community Health, Cardiovascular Section, University of Milan, 20122 Milan, Italy; 5 Development and Innovation Cardiac Surgery Unit, Department of Cardiovascular Disease, Centro Cardiologico Monzino IRCCS, 20138 Milan, Italy; 6 Unit of Cardiac Surgery and Translational Research, IRCCS Policlinico S. Donato, University of Milan, S.Donato Milanese, 20097 Milan, Italy; alessandro.parolari@grupposandonato.it

**Keywords:** *S*-thiolation, mercaptoalbumin, albumin, coronary artery bypass graft, oxidative stress

## Abstract

Coronary artery bypass graft (CABG) surgery still represents the gold standard for patients with complex multivessel coronary artery disease. However, graft occlusion still occurs in a significant proportion of CABG conduits, and oxidative stress is currently considered to be a potential contributor. Human serum albumin (HSA) represents the main antioxidant in plasma through its reduced amino acid Cys34, which can efficiently scavenge several oxidants. In a nested case–control study including 36 patients with occluded grafts and 38 age- and sex-matched patients without occlusion, we assessed the levels of the native mercaptoalbumin (HSA-SH) and oxidized thiolated form of albumin (Thio-HSA) in relation with graft occlusion within 5 years after CABG. We found that the plasma level of preoperative HSA-SH was significantly lower in patients with occluded graft at 5 years follow-up than in patients with graft patency. Furthermore, low HSA-SH remained independently associated with graft occlusion even after adjusting for preoperative D-dimer, a well-known marker of activated coagulation recently found to be associated with graft occlusion. In conclusion, the preoperative level of HSA-SH is independently associated with graft occlusion in CABG and represents a measurable and potentially druggable predictor.

## 1. Introduction

According to the US and European guidelines, coronary artery bypass graft (CABG) surgery represents the gold standard of care for patients with complex multivessel coronary artery disease and/or left main disease, diabetes, or reduced left-ventricular function [1,2].

Despite significant improvements in the outcome of patients undergoing CABG procedure, graft occlusion is the ‘Achilles’ heel’ of this procedure, occurring in a significant number of CABG conduits. Biological mechanisms, characteristics of the target vessels, surgical procedure, and type of graft used all are critical factors in determining failure [3,4]. Indeed, many studies have demonstrated that CABG is associated with the activation of different molecular pathways, which lead to a persistent systemic inflammatory state associated with the activation of the hemostatic systems and oxidative perturbations (reviewed in [5]).

Indeed, the increased production of reactive oxygen species (ROS) is considered a potential contributor to the incidence of perioperative or postoperative complications occurring after CABG, causing damage and dysfunction of macromolecules, cells, and tissues by overwhelming the local antioxidant defense mechanisms. Several studies have shown an increase of oxidative stress during the intraoperative period and the very early hours after surgery, with increases in several pro-oxidant markers [5] and a concomitant decrease in the levels of antioxidant molecules (such as tocopherols and reduced glutathione [6,7]).

Beyond the perioperative period, levels of oxidative stress markers at longer periods have not been assessed. To the best of our knowledge, the only study with a follow-up of 1 week after surgery showed that myeloperoxidase levels increased up to the second postoperative day [8].

Human serum albumin (HSA), beyond its crucial physiological functions (i.e., maintenance of the oncotic pressure and microvascular integrity, regulation of metabolic and vascular functions, and transport of biomolecules and drugs) [9], represents the main antioxidant in plasma [10,11]. Albumin exerts its antioxidant activity through its multiple-binding sites and free radical-trapping properties [10,12], with the latter mainly occurring through the presence of the sulfhydryl group of the amino acid Cys34, which can efficiently scavenge several oxidants, including hydroxyl and peroxyl radicals, hydrogen peroxide, and peroxynitrite [10,11,13,14]. The Cys34 sulfhydryl group present on albumin is the largest fraction of all free thiols in plasma (~80%, corresponding to ~500 µmol/L), thus attributing to HSA, due to its abundance in the plasma, a major role as an antioxidant [10]. Indeed, in plasma samples from healthy subjects, the main HSA fraction is represented by mercaptoalbumin, HSA-SH, which contains a reduced free thiol at Cys34, while a small proportion of the Cys34 forms reversible mixed disulfides with low-molecular-weight thiols, generating *S*-thiolated albumin isoforms. As these oxidized isoforms are not present on albumin after its secretion from hepatocytes but occur after being released into the circulation, they may represent potential biomarkers of oxidative stress in human diseases [11,15].

Therefore, in search of biomarkers that may predict graft occlusion and possibly suggest strategies to reduce graft failure, we assessed preoperative and postoperative levels of native and thiolated forms of albumin in relation to graft occlusion evaluated up to 5 years after CABG.

## 2. Materials and Methods

### 2.1. Study Population

In this study, we used samples from an existing plasma biorepository of a cohort of 330 consecutive patients (age 18 to 89 years) enrolled for elective primary surgical myocardial revascularization between November 2006 and February 2010 at Centro Cardiologico Monzino IRCCS (Milan, Italy) (NCT00755248, CAGE Study) [16]. The CAGE study was approved by the Ethical Committee of Centro Cardiologico Monzino IRCCS (approval number: CCMS90/108) and was conducted according to the Declaration of Helsinki. Informed consent was obtained from all the patients. Patients with concomitant surgery, major end-organ dysfunction, known coagulation disorders, serious undercurrent illness or infection, serum creatinine level greater than 2 mg/dL, and atrial fibrillation were excluded, as previously described [16].

**Figure 1:**
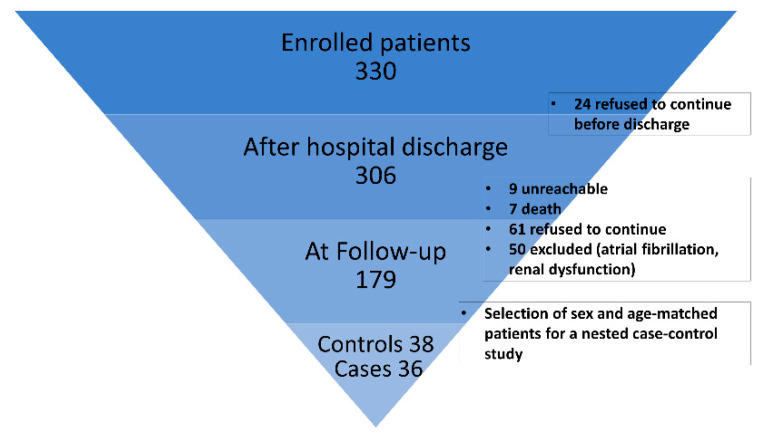
Study enrollment flow chart.

For all patients, postoperative therapy followed guidelines for CAD and surgical myocardial revascularization including always at least single antiplatelet therapy, optimized blood pressure control therapy, and statin. As described in Figure 1, coronary computed tomography angiography (CCTA) and/or coronary angiography were available for 179 patients within a 52 months follow-up for evaluating graft patency.

For this work, a nested case–control study was designed. Suitable samples were available from 36 patients with occluded grafts at 5 years follow-up (cases) and were compared with 38 patients without occlusion (control subjects), frequency-matched for age and sex. Analysis was performed on preoperative plasma samples (T0) and postoperative plasma samples collected at patient discharge (T1), usually between postoperative days 6 and 9 after surgery.

### 2.2. Quantitation of S-Thiolated Albumin by Mass Spectrometry (MS)

The relative composition of albumin isoforms in human plasma samples was evaluated by direct infusion of diluted plasma (500-fold dilution) using the Xevo TQ-S micro triple-quadrupole mass spectrometer coupled with the ACQUITY UPLC^®^ M-Class system (Waters Corporation, Milford, CT, USA), as previously described [17]. After data deconvolution with the MaxEnt1 function on the MassLynx software (Waters Corporation, Milford, CT, USA), HSA-SH, thiolated albumin (+120 ± 2 Da, Thio-HSA), and glycated albumin (Gly-HSA, +160 ± 2 Da) were detected, and their intensities were used to calculate the relative abundances as previously described [13].

### 2.3. Statistical Analysis

Quantitative variables were reported as the mean ± SD or median and interquartile range (IQR) according to their distribution. Spearman’s correlation was used to find monotonic associations between variables. Categorical variables were compared between the two groups using the chi-square test or Fisher’s exact test when appropriate, and quantitative variables were compared using Student’s *t*-test and the Wilcoxon rank sum test when appropriate. Multivariable logistic regression was used to assess whether albumin isoforms measured before surgery were independently associated with graft occlusion. Variables included in the model were selected among potential confounders, i.e., baseline variables that were associated with both the dependent variable (graft occlusion) and the variable under investigation (HSA-SH). The ability of mercaptoalbumin to discriminate between cases and controls was assessed by receiver operating characteristic (ROC) curve analysis. Net reclassification improvement (NRI) was used to check the improvement in risk prediction by adding albumin isoforms to the reference model including D-dimer, logistic EuroSCORE, and extracorporeal circulation (ECC) time. Three risk categories were defined as low (0–33%), middle (33–66%), and high (66–100%). SAS statistical software version 9.4 (SAS Institute, Cary, NC, USA) was used for all analyses, and a *p*-value < 0.05 was considered statistically significant.

## 3. Results

### 3.1. Characteristics of the Study Participants and Study Workflow

This study was performed on a cohort of 74 sex- and age-matched patients, selected from the CAGE study population divided into two groups according to graft patency at 5 years follow-up. We analyzed a total of 36 patients with graft occlusion (cases) and 38 patients with graft patency (controls), with no significant differences between the two groups in terms of age, sex, risk factors, graft vessel origin, and medications (Table 1).

### 3.2. Albumin Isoforms Differences between Occluded and Patent Grafts

Albumin isoforms were analyzed in plasma samples collected before surgery (T0) in cases vs. controls by mass spectrometry, to quantify the percentage of HSA-SH and its main modified isoforms: Thio-HSA and Gly-HSA.

**Figure 2:**
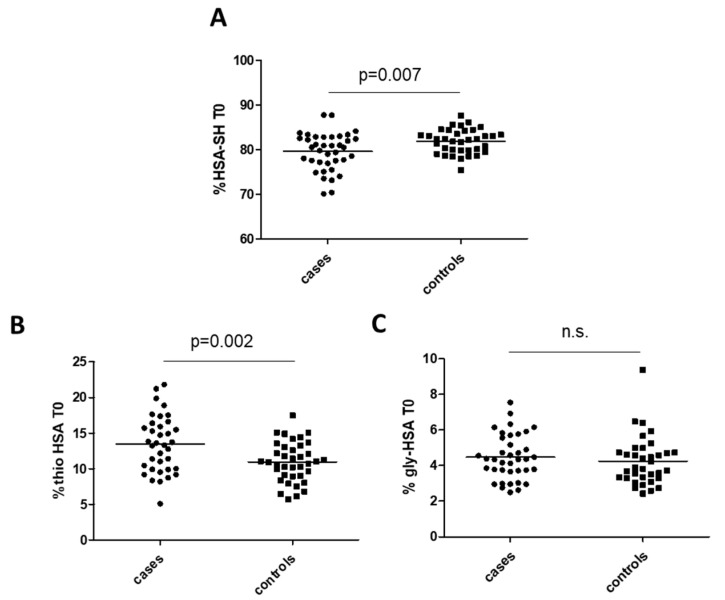
Albumin isoforms in cases and controls: (**A**) mercaptoalbumin (HSA-SH), (**B**) thiolated albumin (Thio-HSA), and (**C**) glycated albumin (Gly-HSA). *p*-Values are from Student’s *t*-test. n.s. means not significant.

At univariable analysis, preoperative HSA-SH resulted significantly lower in cases with occluded graft at 5 years follow-up than in controls (79.6% ± 4.2% and 82.1% ± 2.7%, mean ± SD, *p* = 0.007 in cases and controls, respectively). On the contrary, Thio-HSA showed an inverse behavior (13.5% ± 3.98% and 10.96% ± 2.78% in cases and controls, respectively, *p* = 0.002), whereas Gly-HSA, another modification often occurring in diabetes as the result of glycation reactions, was not different between the two groups (Figure 2).

In addition, Spearman’s correlation analysis (Table 2) showed significant negative associations between HSA-SH and preoperative plasma D-dimer levels, creatinine, and ECC duration, whereas Thio-HSA was positively associated with these three parameters. HSA-SH showed negative correlations also with logistic EuroSCORE and preoperative fibrinogen. Of note, we found a significant positive correlation between antiplatelet therapy and HSA-SH and a negative correlation with Thio-HSA.

Notably, in a multivariable model, adjusting for preoperative D-dimer plasma levels and logistic EuroSCORE, HSA-SH remained independently and negatively associated with graft occlusion within 5 years. This association was still valid even when including ECC duration as a confounding factor (Table 3). Thus, a 19% reduction in the risk of occlusion for a 1% increase in HSA-SH, independent of the confounders considered, was estimated.

On the other hand, Thio-HSA did not result significantly different between cases and controls after adjustment for the above variables (*p* = 0.0946).

### 3.3. HSA-SH as a Predictor of Graft Occlusion

**Figure 3:**
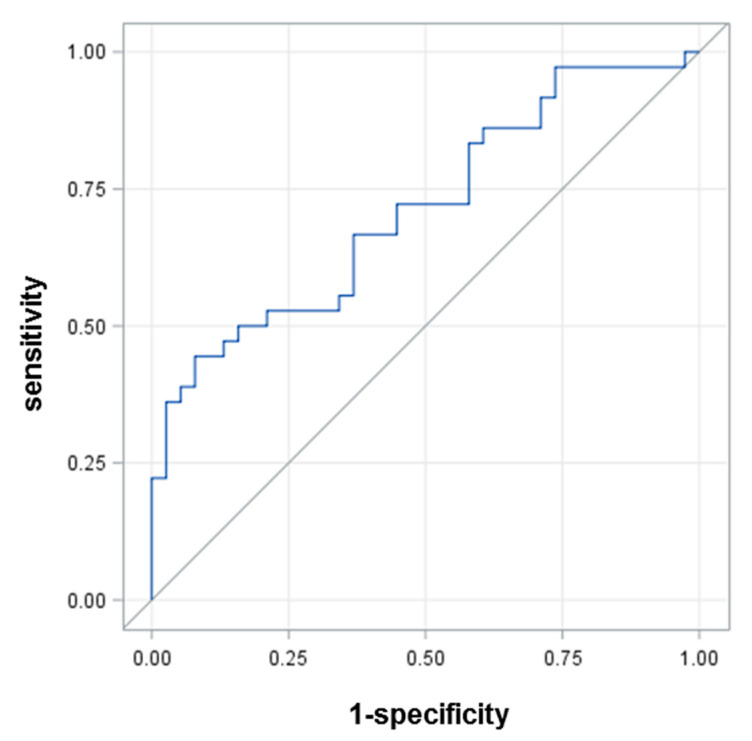
ROC curve for HSA-SH.

To quantify the ability of HSA-SH to predict graft patency at 5 years follow-up, we employed a ROC curve analysis, which revealed an area under curve (AUC) of 0.7126 (Figure 3). This analysis showed that the best HSA-SH cutoff value, which could identify with sufficient accuracy patients who experienced graft occlusion after 5 years, was 81.16% (sensitivity 66.7%, specificity 63.17%).

In addition, to quantify the capacity of HSA-SH to properly classify patients undergoing occlusion, on top of the other variables considered as confounders, we performed a reclassification analysis. On the basis of the NRI, HSA-SH was able to better reclassify 30% of the patients in comparison with a model including D-dimer, logistic EuroSCORE, and duration of ECC (*p* = 0.007, Table S1).

### 3.4. Thio-HSA Increased Significantly after Surgery but Was Not a Predictor of Graft Occlusion

**Figure 4:**
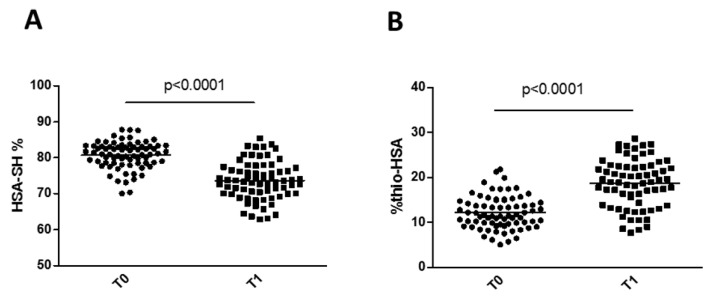
Changes in HSA-SH (**A**) and Thio-HSA (**B**) levels after surgery. *p*-Values are from Student’s *t*-test.

We found a marked increase in the levels of Thio-HSA at patient discharge (T1) compared with preoperative levels, with a concomitant decrease in HSA-SH (Figure 4). Furthermore, HSA-SH was significantly and negatively associated with C-reactive protein (CRP) at discharge (Table 4). However, at discharge, neither Thio-HSA nor HSA-SH levels (expressed as absolute values or delta vs. baseline) correlated with the graft occlusion at 5 years.

## 4. Discussion

This study provides evidence, for the first time, that the preoperative plasma level of HSA-SH was significantly lower in patients with occluded graft than in patients with graft patency at 5 years follow-up, and that it was independently and negatively associated with graft occlusion at 5 years after CABG. Furthermore, low HSA-SH remained independently associated with graft occlusion even after adjusting for preoperative D-dimer, a well-known marker of activated coagulation recently found to be associated with graft occlusion [16]. Furthermore, by assessing the capacity of HSA-SH to properly classify patients undergoing occlusion, on top of other variables considered as confounders, a reclassification analysis based on the NRI revealed that HSA-SH was able to better reclassify 30% of the patients in comparison with a model including D-dimer, logistic EuroSCORE, and duration of ECC (*p* = 0.007).

The novelty of these findings is that it is the quality of albumin, rather than its quantity, that is relevant as a disease biomarker in the cardiovascular setting. Until now, the interest in albumin in the cardiovascular field has been mainly limited to its levels in the bloodstream. Hypoalbuminemia has been associated with poor prognosis in many conditions, such as acute coronary syndrome, heart failure (HF), and in patients undergoing CABG [9,18,19,20]. It has been also demonstrated that patients with preoperative hypoalbuminemia had worse long-term survival after CABG [20]. On the other hand, we recently published new insights into the structural alterations of HSA occurring in the plasma of patients with HF [17]. In this setting, the increase in Thio-HSA and the concomitant decrease in the mercaptoalbumin species correlate with an impairment of the plasma antioxidant activity. Moreover, Thio-HSA levels inversely correlate with peak oxygen uptake (VO_2_), the most objective functional method to assess the cardiopulmonary exercise capacity [17,21]. Furthermore, in HL-1 cardiomyocytes, HSA-SH, but not Thio-HSA, prevented the decrease in cell viability after treatment with hydrogen peroxide [17]. Taken together, these data suggest that albumin thiolation increases in HF patients, provokes alterations in the structure and antioxidant function of HSA, and potentially contributes to HF progression.

After CABG, there is a marked and prolonged activation of many molecular pathways leading to increased inflammation and oxidative stress, hemostasis activation, and unfavorable endothelial milieu. Thus, several biomarkers reflecting the activation of these metabolic systems have been proposed as predictors of early and late graft occlusion [5,16,22,23]. Indeed, the identification of patients who are at risk of graft failure still represents an urgent clinical need and a research challenge.

Considering that oxidized isoforms of albumin are not present in the protein immediately after secretion from the liver, they might represent potential biomarkers of oxidative stress in human diseases [11,15]. Furthermore, as albumin has a half-life of 3 weeks, any modifications of its structure are more stable than the oxidative stress markers usually evaluated so far. For example, a consistent increase in isoprostane 8-iso prostaglandin F_2α_ excretion was observed in CABG patients during surgery, with levels returning to baseline 24 h after surgery. Similarly, malondialdehyde increased significantly during surgery and declined immediately after [6,24]. Of note, in our study, Thio-HSA was still high at patient discharge, thus representing a stable indicator of oxidative stress.

In addition to its role as a potential marker of oxidative stress, it should be emphasized that HSA represents a significant source of antioxidant defense in the plasma, which is constantly exposed to oxidative stress. HSA is involved in scavenging the vast majority of free radicals, thanks to the presence of the free thiol group on Cys34, which constitutes the largest pool of free thiols in circulation and works as a radical scavenger due to its peculiar acidity (pKa = 8.1) and spatial accessibility.

Lastly, we recently showed that Thio-HSA can be restored to HSA-SH by a disulfide-breaking agent, which can, thus, preserve the antioxidant potential of the extracellular milieu [25].

## 5. Conclusions

Although some limitations should be acknowledged such as the relatively small sample size and lack of validation on a larger cohort of patients, together with the fact that we could not precisely establish the timing of graft failure within the follow-up period, we believe that we identified a measurable and druggable predictor of graft occlusion.

## Figures and Tables

**Table 1:** Clinical characteristics of the study population.

Variable	Patent Grafts (*n* = 38)	Occluded Grafts (*n* = 36)	*p*-Value
Age	63.97 ± 2.66 ^#^	63.16 ± 7.97 ^#^	0.56
Male	34 (89.5)	31 (86.1)	0.66
BMI	27.02 ± 3.35 ^#^	25.73 ± 3.03 ^#^	0.09
*Risk factors*			
Diabetes mellitus	13 (34.2)	14 (38.9)	0.68
Hypertension	26 (68.4)	27 (75)	0.54
Hyperlipidemia	32 (84.2)	31 (86.1)	0.82
Current smoker	4 (10.5)	8 (22.2)	0.21 *
*Medications*			
Antiplatelet	28 (77.8)	23 (69.7)	0.45
Hypoglycemic	8 (22.2)	11 (33.3)	0.31
Antihypertensive	32 (88.9)	30 (90.9)	0.99 *
Antiarrhythmic	1 (2.8)	2 (6.1)	0.60 *
Hypolipidemic	25 (69.4)	24 (72.7)	0.77
*Preoperative characteristics*			
LVEF (%)	61.95 ± 6.21 ^#^	54.89 ± 11.26 ^#^	0.001
Additive EuroSCORE	2 (1; 3) ^§^	3 (1; 4) ^§^	0.004
Logistic EuroSCORE	1.3 (1.1; 1.8) ^§^	1.7 (1.3; 3.1) ^§^	0.02
D-dimer T0 (ng/mL)	606.1 (366.11; 976.05) ^§^	796.66 (476.81; 1224.33) ^§^	0.039
CRP T0 (mg/L)	2.29 (1.08; 4.42) ^§^	2.89 (1.29; 7.81) ^§^	0.22
Fibrinogen T0 (mg/dL)	400.18 (356.19; 422.67) ^§^	417.7 (365.85; 461.01) ^§^	0.12
Creatinine T0 (mg/dL)	1.03 ± 0.2 ^#^	1.07 ± 0.23 ^#^	0.43
Diseased coronary vessels (*n*)	2.63 ± 0.61 ^#^	2.91 ± 0.29 ^#^	0.016
Bypass grafts (*n*)	2.68 ± 0.74 ^#^	3.28 ± 0.74 ^#^	0.002 **
Anastomoses (*n*)	2.97 ± 0.88 ^#^	3.6 ± 0.9 ^#^	0.01 **
*Surgery parameters*			
GSV use	34 (89.5)	36 (100)	0.11 *
LITA use	38 (100)	36 (100)	-
RITA use	5 (13.2)	7 (19.4)	0.47
Radial artery use	2 (5.3)	2 (5.6)	0.99 *
Surgery time (h)	4 (3.5; 5) ^§^	4.48 (4; 5) ^§^	0.03
ECC time (min)	85.5 (70; 105) ^§^	107.5 (87.5; 127.5) ^§^	0.001
Clamp time (min)	63 (45; 76) ^§^	75.5 (61.5; 88.5) ^§^	0.004

T0, before surgery; BMI, body mass index; CRP, C-reactive protein; LVEF, left-ventricular ejection fraction; GSV, greater saphenous vein; LITA, left internal thoracic artery; RITA, right internal thoracic artery; ECC, extracorporeal circulation; Continuous data are reported as the mean ± SD ^#^ or median and interquartile range (IQR) ^§^. Categorical variables are expressed as the number and percentage, *n* (%). * Fisher’s exact test. ** Wilcoxon rank sum test.

**Table 2:** Spearman correlations between preoperative albumin isoforms and baseline clinical characteristics.

Variable	HSA-SH T0		Thio-HSA T0	
	*R*	*p*-Value	*R*	*p*-Value
Age	−0.083	0.4682	0.067	0.5594
Sex	−0.029	0.8013	−0.002	0.9844
BMI	0.015	0.8934	0.013	0.9106
*Risk factors*				
Diabetes	0.113	0.3267	−0.235	0.0383
Hypertension	−0.038	0.7414	0.095	0.4085
Hypercholesterolemia	0.123	0.2829	−0.084	0.4666
Smoke	−0.109	0.341	0.06	0.5997
*Medications*				
Antiplatelet	0.261	0.0255	−0.269	0.0216
Hypoglycemic	0.078	0.5143	−0.204	0.0835
Antihypertensive	−0.104	0.3823	0.254	0.0302
Antiarrhythmic	−0.043	0.7206	0.043	0.7206
Hypolipidemic	0.027	0.8187	−0.016	0.8945
*Preoperative * *characteristics*				
LVEF	0.041	0.7187	0.012	0.914
Additive EuroSCORE	−0.214	0.0612	0.186	0.1059
Logistic EuroSCORE	−0.236	0.039	0.199	0.0834
D-dimer T0	−0.324	0.0038	0.241	0.0332
CRP T0	−0.197	0.0852	0.184	0.1087
Fibrinogen T0	−0.25	0.0272	0.211	0.0641
Creatinine T0	−0.346	0.0019	0.302	0.0072
Diseased coronary vessels	0.017	0.8908	−0.061	0.6164
*Intraoperative * *characteristics*				
GSV use	0.034	0.7695	0.01	0.9276
RITA use	0.147	0.1997	−0.18	0.115
RAD use	−0.271	0.0164	0.142	0.215
ECC time	−0.233	0.0459	0.293	0.0113

T0, before surgery; BMI, body mass index; CRP, C-reactive protein; LVEF, left-ventricular ejection fraction; GSV, greater saphenous vein; RITA, right internal thoracic artery; ECC, extracorporeal circulation; RAD, radial artery.

**Table 3:** Logistic models for HSA-SH association with graft occlusion at 5 years follow-up.

Variable	ODDS RATIO	95% Wald		*p*-Value
		Confidence Limits		
*Model A*				
HSA-SH T0	0.847	0.733	0.978	0.0236
* D-dimer T0	1.400	0.763	2.588	0.2747
Logistic EuroSCORE	1.726	1.027	2.902	0.0393
*Model B*				
HSA-SH T0	0.81	0.665	0.988	0.0372
* D-dimer T0	1.453	0.757	2.79	0.2613
ECC time	1.025	1.003	1.048	0.0278
Logistic EuroSCORE	1.72	0.898	3.298	0.1022

Model A considers preoperative plasma levels of D-dimer and logistic EuroSCORE as confounding factors. Model B also includes the time of ECC during surgery. ECC, extracorporeal circulation. * The D-dimer odds ratio was computed for one standard deviation increase (~500 ng/mL).

**Table 4:** Spearman correlation for mercaptoalbumin (HSA-SH) and thiolated albumin (Thio-HSA) after surgery.

Variable	HSA-SH T1		Thio-HSA T1	
	*R*	*p*-Value	*R*	*p*-Value
D-dimer T1	−0.21294	0.0666	0.20649	0.0755
CRP T1	−0.26765	0.0203	0.13935	0.2331
Graft occlusion	−0.03696	0.7529	0.05297	0.6517

CRP, C-reactive protein.

## Data Availability

Data collected in the study will be made available using the data repository Zenodo (https://zenodo.org/), doi:10.5281/zenodo.6405086, accessed on 10 February 2022.

## Supplementary Materials

The following supporting information can be downloaded at https://www.mdpi.com/article/10.3390/antiox11040702/s1: Table S1. Results from NRI analysis.
