# Supplementary material for: Mercaptoalbumin Is Associated with Graft Patency in Patients Undergoing Coronary Artery Bypass Grafting
Source: Antioxidants (Basel). 2022 Apr 2;11(4):702. doi: 10.3390/antiox11040702 (PMC9029960; doi:10.3390/antiox11040702)
Supplement: Supplementary file 1 [file antioxidants-11-00702-s001.zip › antioxidants-1613231-supplementary.pdf]

Table S1. Results from NRI analysis. Simple-model included D-Dimer, logistic euroscore and ECC time. Added model included also HSA-SH. NRI is the algebraic sum of the percent of events and non-events correctly reclassified minus those wrongly reclassified

Events

	Added model			
**Simple model**	**<33%**	**33-66%**	**>66%**	**Total**
**<33%**	3 (8.8)	2 (5.9)	0 (0)	5 (14.7)
**33-66%**	1 (2.9)	10 (29.4)	4 (11.8)	15 (44.1)
**>66%**	0 (0)	0 (0)	14 (41.2)	14 (41.2)
**Total**	4 (11.8)	12 (35.3)	18 (52.9)	34 (100)

Non events

	Added model			
**Simple model**	**<33%**	**33-66%**	**>66%**	**Total**
**<33%**	16 (42.1)	1 (2.6)	0 (0)	17 (44.7)
**33-66%**	6 (15.8)	10 (26.3)	1 (2.6)	17 (44.7)
**>66%**	0 (0)	2 (5.3)	2 (5.3)	4 (10.5)
**Total**	22 (57.9)	13 (34.2)	3 (7.9)	38 (100)

	**Reclassified**		**Net**	**NRI**
	**higher**	**lower**		
**Events**	6 (17.6%)	1 (2.9%)	14.7%	
**non events**	2 (5%)	8 (21%)	15.8%	30.5%
